# Relative Effectiveness of High‐Dose vs. Standard‐Dose Influenza Vaccines in Preventing Hospitalizations: A National Retrospective Cohort Study in France, 2022/2023 Season

**DOI:** 10.1111/irv.70193

**Published:** 2025-11-16

**Authors:** Hélène Bricout, Marie‐Cécile Levant, Pascal Crépey, Gaëtan Gavazzi, Jacques Gaillat, Marine Dufournet, Nada Assi, Benjamin Grenier, Fanny Raguideau, Fabienne Péretz, Camille Salamand, Anne Mosnier, Laurence Watier, Odile Launay, Matthew M. Loiacono

**Affiliations:** ^1^ Medical Department Sanofi Vaccines Lyon France; ^2^ Ecole des Hautes Études en Santé Publique, CNRS Université de Rennes, ARENES, UMR 6051, Recherche sur les Services et le Management en Santé, Inserm U 1309 Rennes France; ^3^ CHU Grenoble Alpes Service Universitaire de Gériatrie Clinique, CS 10217 Grenoble France; ^4^ Laboratoire T‐Raig TIMC‐IMAG CNRS 5525 Université Grenoble‐Alpes La Tronche France; ^5^ Service de Maladies Infectieuses Centre Hospitalier Annecy Genevois Annecy France; ^6^ Biostatistics Department Sanofi Vaccines Lyon France; ^7^ HEVA, Pôle Statistiques Lyon France; ^8^ HEVA, Pôle Epidémiologie Lyon France; ^9^ Abelia Science Saint‐Georges‐sur‐Baulche France; ^10^ Open Rome Paris France; ^11^ Epidemiology and Modeling of Bacterial Escape to Antimicrobials, Institut Pasteur Paris France; ^12^ Inserm CIC 1417, Assistance Publique Hôpitaux de Paris, Hôpital Cochin Université Paris Cité Paris France; ^13^ Medical Evidence Generation Sanofi Vaccines Swiftwater Pennsylvania USA

**Keywords:** aged, factual databases, France, hospitalization, influenza vaccines, medical informatics, vaccine effectiveness

## Abstract

**Background:**

A French cohort study (2021/2022 influenza season) found the high‐dose influenza vaccine (HD) more effective than standard‐dose vaccines (SDs) in preventing influenza‐related hospitalizations in the elderly. The study continued to refine results and validate these findings.

**Methods:**

Data from community‐dwelling 65+ adults who received HD or SD during the 2022/2023 vaccination campaign were extracted from the National Health database. Hospitalizations were recorded from 14 days postvaccination until June 30, 2023. HD and SD recipients were matched using a propensity score. Associations between vaccines and hospitalizations were assessed by estimating incidence rate ratios and converting them to HD vs. SD vaccine relative effectiveness (rVE).

**Results:**

A total of 675,412 HD recipients were matched to 2,701,648 SD recipients. The rVE for influenza‐related hospitalizations was 27.4% [95% CI: 19.8; 34.3]. It ranged from 22.7% [9.8; 33.6] to 33.6% [21.2; 44.0] across age groups, indicating that HD consistently outperformed SDs in preventing influenza‐related hospitalizations, with the highest effect observed in 85+.

**Conclusions:**

Our study is the first to publish rVE data comparing HD and SDs in a real‐world setting in France for the 2022/2023 influenza season. Its findings reaffirm the benefit of HD vs. SDs. HD could help reduce the burden of severe respiratory infections in the elderly.

**Abbreviations**
ad
: associated diagnosis
AIC: Akaike information criterion
ALD: long‐term disease (*Affection Longue Durée*)
CI: confidence interval
COPD: chronic pulmonary obstructive disease
FDep: French deprivation index
FWER: family‐wise error rate
HD: high‐dose influenza vaccine
ICD: International Classification of Diseases 10th Revision
IRR: incidence rate ratio
PD: primary diagnosis
RD: related diagnosis
rVE: relative vaccine effectiveness
SD: standard‐dose influenza vaccine
SFGG: French Geriatric Society (*Société Française de Gériatrie et Gérontologie*)
SNDS: French Health Data System (*Système National Des Données de Santé*)
UTI: urinary tract infections
WHO: World Health Organization

## 1 Introduction

Across most countries, influenza vaccination programs aim to reduce the number of severe cases, complications, and deaths [1]. Amongst these programs, recommendations particularly target high‐risk individuals such as adults aged 60 or 65 and over (65+). The heightened susceptibility to infections and severe outcomes in this population can be explained by immunosenescence, age‐related anatomical and physiological changes, a higher prevalence of comorbidities, and malnutrition [2].

Although effective and essential from the public health perspective, current inactivated standard‐dose influenza vaccines (SDs), which contain 15 μg of hemagglutinin per recommended strain, offer suboptimal protection in older adults, primarily because of immunosenescence and age‐related increased inflammation [3]. Indeed, the protection elicited by the vaccine is lower among 65+ than among younger adults [4]. To enhance immune response and decrease influenza‐related morbidity and mortality, a high‐dose influenza vaccine (HD), containing four times each of the antigens of the SDs, was developed. Initially trivalent and licensed in the United States in 2009, a quadrivalent formulation was developed a decade later and approved for use in the United States, Australia, Canada, and Europe [2]. The HD superior relative efficacy against severe clinical outcomes compared with SDs has been demonstrated in a pivotal randomized controlled study, confirmed by several randomized clinical trials, and consistently found in observational studies [4, 5, 6, 7]. According to a recent meta‐analysis [7], HD reduced hospitalizations because of pneumonia or influenza (P/I) by 23.5% [95% confidence interval (95% CI): 12.3; 33.2] compared with SDs in 65+.

In France, HD was used for the first time during the 2020/2021 influenza season. In May 2020, the French Health Authority additionally recommended its use in 65+ under the same conditions of distribution, administration, and reimbursement as SDs. In practice, before the start of each influenza season, high‐risk individuals are provided with a voucher to get their influenza vaccine free of charge at a pharmacy. The vaccine is then administered by the pharmacist or another healthcare professional authorized to vaccinate, typically the family practitioner or a nurse. In line with the recommendations of the French Health Authority, any of the available influenza vaccines can be used for 65+; however, preferential use of HD for 65+ is recommended by the French Geriatric Society (SFGG) [8, 9].

Using the French national administrative healthcare data collected from the start of the 2021/2022 influenza vaccination campaign to June 30, 2022, we previously conducted an observational study called DRIVEN [10]. This study confirmed that HD provided better protection than SDs across more than 400,000 HD recipients and 1,600,000 SD recipients (rVE: 23.3% [95% CI: 8.4; 35.8]). The study continued during the 2022/2023 season to obtain more accurate results, given the increased use of HD between the 2021/2022 and 2022/2023 seasons, and to confirm the findings during a different influenza season, considering potential epidemiological changes. The objectives of the present study were thus to estimate the rVE of HD vs. SD in preventing hospitalizations due to influenza and noninfluenza‐specific causes overall (primary objective), as well as by age group (secondary objective), among 65+, during the 2022/2023 season, in France. We anticipated the results from the 2022/2023 season would confirm those of the 2021/2022 season, improving robustness and precision owing to more individuals vaccinated with HD.

## 2 Methods

### 2.1 Study Design

The method was identical to that used for the 2021/2022 influenza vaccination campaign (Season 1) [10]. Briefly, DRIVEN Season 2 was an observational retrospective cohort study, based on the National Health Data System (SNDS). It was designed to assess the rVE of HD vs. SDs for hospitalization outcomes for the 2022/2023 influenza season. Vaccine type (HD or SD) was identified using medication codes (Table S1).

### 2.2 Study Population

The study cohort included all 65+ (on the day of vaccine dispensing) with a record of influenza vaccine dispensed at a pharmacy during the official influenza vaccination campaign period (Oct.18, 2022/Mar. 31, 2023). Individuals remained in the study cohort from the day of influenza vaccine dispensing up to June 30, 2023, or the day of admission to medico‐social housing or a nursing home, or death.

### 2.3 Data Collection

The study used data from the SNDS, part of the National Health Insurance system. The SNDS encompasses anonymous, individual‐level data for all healthcare claims for more than 99% of the population residing in France, regardless of their insurance scheme, i.e., close to 68 million people.

Baseline demographics, underlying diseases/medical history (medical conditions), and previous treatments and vaccinations were captured from Sept. 1, 2017, to Mar. 31, 2023, using both community and hospital‐based data from SNDS. Hospitalizations related to influenza or other causes were collected from 14 days after the index date (i.e., presumed day of the start of the vaccine protection) to the end of follow‐up. The index date was defined as the date of the claim for influenza vaccine reimbursement (= dispensing date); it was used as a proxy for influenza vaccination. Fourteen days allow sufficient time for vaccine‐induced immunity to develop [11]. By convention, individuals were referred to as HD or SD recipients.

### 2.4 Primary Outcomes

Primary outcomes were hospitalizations for influenza and noninfluenza‐specific causes (pneumonia, P/I, respiratory diseases, or cardiovascular or cardiorespiratory events). Hospitalizations were ascertained by the International Classification of Diseases 10th Revision (ICD‐10) discharge diagnosis code (Table S1). The ICD‐10 discharge diagnosis code in the database could be noted as primary (PD), related (RD), or associated (ad).

### 2.5 Matching

Since the study relied on retrospective database analysis with routine treatment allocation (no randomization), a treatment selection or indication bias could exist. To adjust for potential confounding effects, each HD recipient was matched to four SD recipients using a propensity score with an exact constraint for sex, age group (65–75, > 75–85, and > 85 years), geographical region, and week of vaccine dispensing. The propensity score was computed using a logistic regression model including other covariables: medical variables such as previous visits to family practitioners or hospitalizations; vaccine histories such as previous influenza, pneumococcal, or COVID‐19 vaccinations; social characteristics such as quintiles of the French deprivation index (FDep); and underlying diseases such as diabetes, obesity, malnutrition, or chronic obstructive pulmonary disease (COPD) at dispensing date [10].

### 2.6 Statistical Methods

To estimate the association between vaccination with HD or SD and hospitalization outcome, incidence rate ratios (IRRs) were calculated and converted to rVE. Poisson models, negative binomial model, and their zero‐inflated counterparts were used to estimate IRRs along with their corresponding 95% CI [12]. The model with the lowest Akaike information criterion (AIC) was chosen [13]. Models included an offset for the log of the follow‐up time, which allowed a rate model to be computed. The rVE was computed as ([1 − IRR)] * 100), with its corresponding 95% CI by Taylor series variance approximation [14, 15].

IRRs and rVEs were calculated for hospitalizations for influenza, pneumonia, P/I, respiratory events, cardiorespiratory events, and cardiovascular events in the whole population and hospitalizations for influenza, pneumonia, and P/I per age group.

The main analysis was performed on the matched population using the PD code to ascertain hospitalization diagnosis. Hospitalizations with a discharge diagnosis code associated with COVID‐19 were excluded from this main analysis (COVID‐19 exclusion). Sensitivity analyses were performed with alternative outcome definitions: i.e., less specific ICD‐10 discharge diagnosis code (i.e., PD, RD, or ad) or hospitalizations (i.e., COVID‐19 included). To increase the specificity of the outcomes, an analysis was performed considering only hospitalizations that occurred from 4 weeks before to 4 weeks after the week of the peak incidence (9 weeks) for the 2022/2023 influenza season: i.e., from Nov. 21, 2022, to Jan. 22, 2023, as the peak incidence occurred during Week 51.

A falsification analysis was performed with negative control outcomes (i.e., hospitalizations for urinary tract infections (UTIs), cataract surgery, or erysipelas) to assess any potential residual bias due to unmeasured confounding. Stability analyses (including classical multivariable analysis) were conducted to assess the robustness and consistency of the results (Data S1).

Statistical analysis was performed using SAS Software, Version 9.4 (SAS Institute Inc., Cary, NC, USA). To account for the multiplicity of testing, the results of the main and sensitivity analyses were controlled using the family‐wise error rate (FWER) through the Bonferroni–Holm procedure. This method provided tight control over the probability of making one or more Type I errors (false positives), and the corrected *p*‐values were presented accordingly. For the falsification analysis, the results were interpreted with an unadjusted level of Type I error. The statistical significance threshold was set at 0.05.

## 3 Results

### 3.1 Study Population and Vaccine Exposure

**FIGURE 1:**
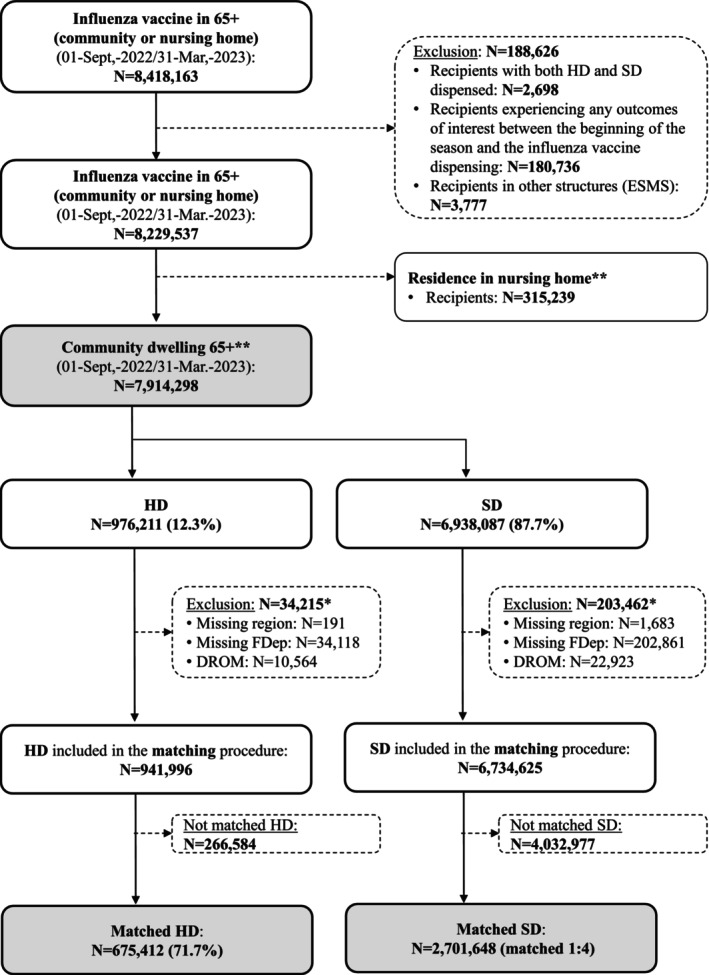
Study flow chart. *Details are nonexclusive: For example, recipients could have been excluded for a missing FDep and because they resided in the DROM. **On the date, the vaccine is dispensed 65+: aged 65 and over; DROM: abbreviation for overseas departments and regions of France; ESMS: other social health‐care institutions; FDep: French deprivation index; HD: high‐dose influenza vaccine (or cohort); N: number of recipients; SD: standard‐dose influenza vaccine (or cohort).

Of the 8,418,163 recipients identified in the database for the 2022/2023 influenza vaccination campaign, 7,914,298 were 65+ living in the community. Of these, 976,211 (12%) were HD recipients and 6,938,087 (88%) were SD recipients. After matching (1:4), 675,412 HD recipients and 2,701,648 SD recipients constituted the analysis population (Figure 1). The rate of matching was 72% in the HD cohort: 81% in the 65–75; 68% in the 75–85; and 61% for the 85+. In the SD cohort, 36% of the 65–75, 42% of the 75–85, and 48% of the 85+ were matched with HD recipients. The main characteristics for matched recipients are presented in Table 1.

**TABLE 1:** Main baseline characteristics of each cohort (matched recipients only, *N* = 3,377,060).

Characteristics	HD cohort	SD cohort
Number of individuals	*N* = 675,412	*N* = 2,701,648
Age (years), mean (SD)	76.83 (7.68)	76.78 (7.73)
Age (groups), *n* (%)		
	302,005 (44.71)	1,208,020 (44.71)
	265,528 (39.31)	1,062,112 (39.31)
	107,879 (15.97)	431,516 (15.97)
Sex: women, *n* (%)	371,550 (55.01)	1,486,200 (55.01)
Reasons for end of follow‐up, *n* (%)		
Admission to a medico‐social housing (other than NH)	96 (0.01)	394 (0.01)
Admission to NH	3247 (0.48)	12,308 (0.46)
Death	12,354 (1.83)	45,393 (1.68)
End of follow‐up	659,715 (97.68)	2,643,553 (97.85)
Health‐seeking behaviors proxy		
All‐cause hospitalization in the past 12 months, mean (SD)	0.14 (0.99)	0.13 (0.99)
GP visits in the past 12 months, mean (SD)	5.80 (4.42)	5.73 (4.38)
Influenza vaccination at the pharmacy, *n* (%)	326,738 (48.38)	1,300,091 (48.12)
Influenza vaccination during the previous season, *n* (%)	620,982 (91.94)	2,478,557 (91.74)
COVID‐19 vaccinated^a^, *n* (%)	657,801 (97.39)	2,632,920 (97.46)
Pneumococcal vaccination in the previous 5 years, *n* (%)	82,789 (12.26)	323,832 (11.99)
Medical diseases or conditions reported during the 5 years preceding the index date, *n* (%)		
Diabetes	141,985 (21.02)	558,016 (20.65)
Obesity and/or history of obesity surgery	55,830 (8.27)	216,516 (8.01)
Undernourishment/or history of undernourishment	40,626 (6.01)	157,605 (5.83)
COPD/asthma	82,238 (12.18)	320,773 (11.87)
Dementia	18,039 (2.67)	67,799 (2.51)
Cardiovascular diseases	188,455 (27.90)	732,952 (27.13)
Immunocompromised individuals	128,518 (19.03)	499,107 (18.47)
Chronic liver disease	10,683 (1.58)	41,777 (1.55)
Terminal chronic kidney failure	3242 (0.48)	12,553 (0.46)
Number of medical diseases or conditions reported during the 5 years preceding the index date, *n* (%)		
None	302,797 (44.83)	1,250,066 (46.27)
1	217,462 (32.20)	849,080 (31.43)
2	97,773 (14.48)	379,766 (14.06)
3	38,282 (5.67)	148,363 (5.49)
4	13,458 (1.99)	52,144 (1.93)
5	4200 (0.62)	16,419 (0.61)
6+	1440 (0.21)	5810 (0.22)
Access to care (LPA, quintiles), *n* (%)		
Missing data	2178 (0.32)	10,522 (0.39)
Q1, Individuals living in the poorest municipalities	136,258 (20.17)	532,761 (19.72)
Q2	129,386 (19.16)	565,874 (20.95)
Q3	140,781 (20.84)	540,679 (20.01)
Q4	140,774 (20.84)	549,137 (20.33)
Q5, Individuals living in the wealthiest municipalities	126,035 (18.66)	502,675 (18.61)
Precariousness index (FDep), *n* (%)		
Q1, Individuals living in the least disadvantaged municipalities	134,033 (19.84)	534,373 (19.78)
Q2	133,609 (19.78)	535,348 (19.82)
Q3	138,963 (20.57)	560,556 (20.75)
Q4	140,353 (20.78)	558,286 (20.66)
Q5, Individuals living in the most disadvantaged municipalities	128,454 (19.02)	513,085 (18.99)

*Note:* It reflects the COVID‐19 vaccination status of each patient at index date following current guidelines (it can refer to a single dose, 2, or 3, depending on the individual's eligibility.

Abbreviations: COPD: chronic obstructive pulmonary disease; FDep: French deprivation index; HD: high‐dose influenza vaccine; LPA: local potential accessibility; NH: nursing home; SD: standard deviation; SD: standard dose influenza vaccine.

^a^ ‘COVID‐19 vaccinated’ is a variable identified as such within the database.

Before matching (*N* = 7,914,928), the two cohorts differed for several baseline characteristics (Table S2). HD recipients were older (median: 77 vs. 75 years); more frequently presented with at least one specific long‐term disease (ALD) (52% vs. 48%); more frequently received at least one influenza vaccine administration during the previous 5 years (97% vs. 94%); and had a poorer outcome at the end of the study (2.1% vs. 1.5% died). Most HD doses were dispensed within the first 4 weeks of the influenza vaccination campaign for HD (81%) but within the first 7 weeks for SD doses (88%) (Figure S1). Compared with 75–85 and 65–75 recipients, recipients aged 85+ more frequently presented with at least one ALD (61% vs. 52% in the 75–85 group and 42% in the 65–75 group); more frequently received at least one influenza vaccine administration during the previous 5 years (98% vs. 98% and 91%, respectively); more frequently provided with the doses of influenza vaccine within the first 4 weeks of the vaccination campaign (64% vs. 58% and 54%); and had a poorer outcome at the end of the study (5% vs. 1.4% and 0.6% died) (Table S3).

After matching (*N* = 3,377,060), the two cohorts were overall well balanced (Table S4). Standardized differences ranged between −0.1 and 0.1. However, there was a slight trend toward a marginally better health status in SD than HD recipients: The percentages of recipients having or having experienced medical conditions within the last 5 years were slightly higher in the HD than in the SD cohort as were the percentages of recipients who died or were admitted to a nursing home during the study.

Compared with unmatched HD recipients, matched HD recipients were younger (median: 76 vs. 80 years) and less frequently reported having specific long‐term diseases (50.5% vs. 56%). They exhibited a lower prevalence of medical conditions in the previous 5 years (55% vs. 62%) and a reduced risk of death during the study period (mortality rate, 0.5% vs. 0.8%). They were more frequently located in the South of France (AURA, PACA, Occitanie, Nouvelle Aquitaine: 43% vs. 22%). In the SD cohort, matched recipients were older (median: 76 vs. 74 years) and more frequently reported having specific long‐term diseases (49% vs. 47%) than unmatched recipients. They exhibited a higher prevalence of medical conditions in the previous 5 years (54% vs. 52%) and an increased risk of mortality during the study period (0.5% vs. 0.3%). The discrepancies between matched and unmatched recipients were more pronounced in the HD than in the SD cohort (Table S4).

### 3.2 rVE Estimates and IRR

The influenza‐related hospitalization rates were 125.8 per 100,000 person‐years and 173.4 per 100,000 person‐years in the HD and SD cohort, respectively, converting to an estimated rVE of 27.4% [19.8; 34.3]. The estimated rVE ranged from 19.1% when the ICD‐10 discharge diagnosis code was less specific (i.e., PD, RD, or ad) to 29.9% when the analysis was restricted around the seasonal peak (Table 2).

**TABLE 2:** Incidence rate ratios and relative vaccine effectiveness of HD compared with SDs.

Vaccine group	Hospitalization rate per 100,000 person‐years (95% CI)	IRR (95% CI)	rVE (%) (95% CI)	*p*
Main analysis				
HD	125.76 (115.37–137.10)	0.73 [0.66; 0.80]	27.39 [19.79; 34.27]	< 0.0001
SD	173.36 (167.11–179.85)	1.00		
Sensitivity analysis: PD, RD, ad				
HD	210.82 (197.23–225.35)	0.81 [0.75; 0.88]	19.07 [12.37; 25.27]	< 0.0001
SD	260.69 (252.99–268.61)	1.00		
Sensitivity analysis: With a COVID‐19 code				
HD	127.96 (117.47–139.38)	0.73 [0.66; 0.80]	27.14 [19.57; 33.99]	< 0.0001
SD	175.80 (169.50–182.33)	1.00		
Sensitivity analysis: At the peak of the season				
HD	91.40 (82.60–101.13)	0.70 [0.62; 0.79]	29.91 [21.22; 37.65]	< 0.0001
SD	130.68 (125.26–136.33)	1.00		

Abbreviations: CI: confidence interval; HD: high‐dose influenza vaccine; IRR: incidence rate ratio; rVE: relative vaccine effectiveness; SD: standard‐dose influenza vaccine.

**FIGURE 2:**
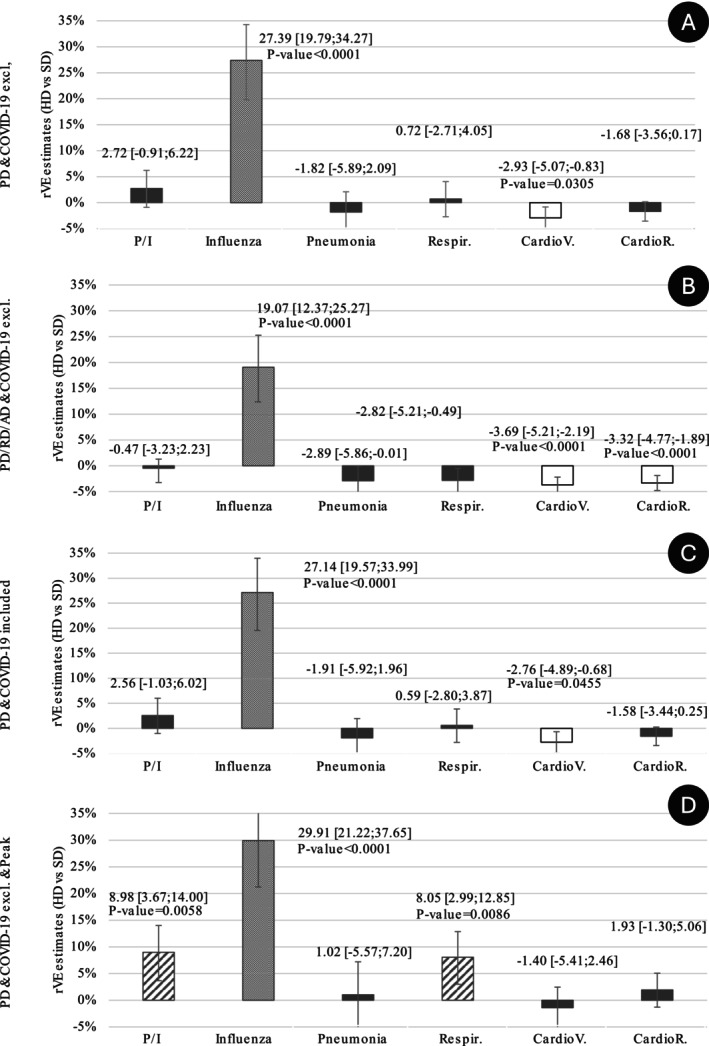
Relative vaccine effectiveness (rVE) of HD compared with SDs during the 2022/2023 influenza season. ad: associated diagnosis; CardioR: cardiorespiratory event; CardioV: cardiovascular event; excl.: excluded; HD: high‐dose influenza vaccine; PD: primary diagnosis; P/I: pneumonia or influenza; RD: related diagnosis; Respir: respiratory event; SD: standard‐dose influenza vaccine. The ICD‐10 discharge diagnosis code in the database could be noted as primary (PD), related (RD), or associated (ad). Values are odds ratios (OR) with 95% confidence intervals [95% CI]. Only *p*‐values below 0.05 after Bonferroni–Holm correction are provided.

Noninfluenza‐specific hospitalizations (i.e., due to pneumonia, P/I, respiratory diseases, cardiovascular events, or cardiorespiratory events) are shown in Figure 2. In the primary analysis (Figure 2A), no significant differences were observed for most noninfluenza‐specific endpoints, albeit for cardiovascular hospitalizations, where event rates were higher in the HD than the SD cohort. Similar trends were observed in the sensitivity analysis (Figure 2B,C). During the peak season, statistically significant differences were observed for hospitalizations for P/I and hospitalizations for respiratory diseases (Figure 2D).

The estimated rVE for influenza‐related hospitalizations varied from 22.6% to 33.6% across age groups, indicating HD yielded better protection against influenza‐related hospitalizations compared with SDs, regardless of age (Table 3). The rVE was particularly high in the 85+ group. In this population, the influenza‐related hospitalization rate was 279.9 per 100,000 person‐years and 419.8 per 100,000 person‐years in the HD and SD cohort, respectively, translating to an estimated rVE of 33.6%. Similar results were found at the peak of the season (85+, rVE = 31.7%) or after the inclusion of hospitalizations with COVID‐19 code (rVE = 32.9%). When the ICD‐10 discharge diagnosis code was less specific, the rVE was lower but remained above that observed for the whole population (rVE = 25.0%) (Table 3).

**TABLE 3:** Incidence rate ratios and relative vaccine effectiveness of HD compared with SDs according to the age group.

	Age group	Vaccine group	Hospitalization rate per 100,000 person‐years (95% CI)	IRR (95% CI)	rVE (%) (95% CI)	*p*
Main analysis	< 75 years	HD	63.45 (52.97–76.00)	0.74 [0.60; 0.91]	25.79 [8.88; 39.56]	0.0044
		SD	85.88 (79.48–92.81)	1.00		
	75–85 years	HD	137.20 (120.25–156.53)	0.77 [0.66; 0.90]	22.65 [9.84; 33.64]	0.0010
		SD	177.45 (167.46–188.04)	1.00	.	
	> 85 years	HD	279.90 (241.56–324.33)	0.66 [0.56; 0.79]	33.55 [21.19; 43.98]	< 0.0001
		SD	419.76 (395.28–445.75)	1.00	.	
Sensitivity analysis:	< 75 years	HD	111.84 (97.63–128.12)	0.82 [0.70; 0.96]	17.89 [3.53; 30.11]	0.0165
PD, RD, ad		SD	137.49 (129.32–146.18)	1.00	.	
	75–85 years	HD	223.49 (201.55–247.81)	0.86 [0.76; 0.97]	14.07 [2.69; 24.11]	0.0168
		SD	260.13 (247.97–272.88)	1.00	.	
	> 85 years	HD	469.67 (419.18–526.24)	0.75 [0.66; 0.86]	25.03 [14.19; 34.49]	< 0.0001
		SD	623.71 (593.72–655.23)	1.00	.	
Sensitivity analysis:	< 75 years	HD	63.45 (52.97–76.00)	0.73 [0.59; 0.90]	27.05 [10.45; 40.57]	0.0026
With a COVID‐19 code		SD	87.36 (80.90–94.34)	1.00	.	
	75–85 years	HD	140.30 (123.15–159.84)	0.78 [0.67; 0.91]	22.00 [9.22; 32.98]	0.0013
		SD	179.93 (169.87–190.59)	1.00	.	
	> 85 years	HD	286.23 (247.43–331.12)	0.67 [0.57; 0.80]	32.86 [20.49; 43.31]	< 0.0001
		SD	424.88 (400.25–451.03)	1.00	.	
Sensitivity analysis:	< 75 years	HD	49.47 (40.33–60.68)	0.76 [0.60; 0.96]	23.98 [3.86; 39.90]	0.0221
At the peak of the season		SD	65.45 (59.89–71.53)	1.00	.	
	75–85 years	HD	92.50 (78.78–108.61)	0.68 [0.57; 0.82]	31.72 [17.89; 43.23]	< 0.0001
		SD	135.57 (126.87–144.86)	1.00	.	
	> 85 years	HD	211.90 (178.90–251.00)	0.68 [0.56; 0.83]	31.72 [16.73; 44.01]	0.0002
		SD	309.69 (288.76–332.13)	1.00	.	

Abbreviations: CI: confidence interval; HD: high‐dose influenza vaccine; IRR: incidence rate ratio; rVE: relative vaccine effectiveness; SD: standard‐dose influenza vaccine.

The IRRs were 1.04 [1.00; 1.07] against hospitalizations related to UTIs, 1.02 [1.00; 1.03] against hospitalizations for cataract surgery, and 1.00 [0.93; 1.08] against hospitalizations due to erysipelas, converting to rVEs of −3.5 [−7.0; −0.15] (*p* = 0.040), −1.7 [−3.2; −0.2] (*p* = 0.025), and −0.3 [−7.9; 6.9] (*p* = 0.944), respectively, suggesting the existence of residual unmeasured confounding factors.

Considering multivariable analysis in the whole population (i.e., all 65+ recipients, *N* = 7,676,621), the influenza‐related hospitalization rate (DP only, COVID‐19 excluded) was 151.4 [141.7; 161.8] per 100,000 person‐years in the HD full cohort and 154.7 [150.8; 158.6] per 100,000 person‐years for the SD full cohort. Including the same covariables as in the matching procedure, the corresponding estimated adjusted rVE was very close to that obtained after matching: 27.4% [21.2; 33.1] (*p* < 0.001).

## 4 Discussion

The DRIVEN Season 2 results reinforce those of Season 1 [10], where HD was significantly associated with fewer influenza‐related hospitalizations compared with SDs in community‐dwelling 65+: The rVE of the HD was 23.3% [8.4; 35.8] for Season 1 and 27.4% [19.8; 34.3] for Season 2. As with all observational studies, the risk of bias limits the conclusiveness of these findings without prior randomized controlled trials. However, the benefits of HD over SD in preventing influenza have been well established in clinical trials and consistently confirmed in observational studies, lending credibility to the observed reduction in influenza‐related hospitalizations [5, 7].

According to the meta‐analysis by Lee et al. [5], HD significantly decreased the risk of hospitalizations because of P/I (odds ratio [OR] = 0.9 [0.8; 0.9]) or cardiorespiratory diseases (OR = 0.8 [0.8; 0.9]). Moreover, in their meta‐analysis, Skaarup et al. [7] found an rVE of 23.5% [12.3; 33.2] for P/I‐related hospitalizations and 7.3% [4.5; 10] for all‐cause hospitalizations. In our study, HD was not significantly associated with reduced hospitalizations because of noninfluenza‐specific causes (Figure 2). However, benefits for hospitalizations due to P/I or cardiorespiratory events were seen during the influenza season peak. Residual confounding bias may partly explain this discrepancy, as supported by our falsification analysis. For less‐specific endpoints, with relative effects of smaller magnitude, such effects may obscure associations, possibly underestimating HD benefit. However, the benefit of HD on influenza‐related hospitalizations remained robust, increasing confidence in the findings; benefits were consistent regardless of the type of influenza diagnosis (primary, associated, related) and whether COVID‐19 cases were included or excluded. As expected, the highest rVE was observed during the influenza season peak.

Considering influenza‐related hospitalizations, the rVE was consistent with the previous season but also aligned with the results of DiazGranados et al. [4] (23.6%) [−2.4; 43.2]). It surpassed estimates from meta‐analysis by Lee et al. (11.2% [7.4; 14.8]) [4, 5, 10]. Differences between Seasons 1 and 2 were partly due to twice as many HD recipients in 2022/2023 compared with 2021/2022 and to variations in influenza epidemic characteristics in France. The 2021/2022 season, marked by the cocirculation of A(H3N2) and A (H1N1)pdm09, had a late onset (Mar. 2022), short duration (9 weeks), and low severity in 65+. The 2022/2023 season, driven mainly by the A(H3N2) virus, began early (Nov. 2022), lasted longer (19 weeks), and was more severe in 65+ [16, 17]. Given the segmented RNA structure and high mutation rate of influenza viruses, vaccines are regularly updated to reflect antigenic and epidemiological changes. Vaccine composition is reassessed yearly based on global surveillance data, and the World Health Organization (WHO) recommends strains for each hemisphere accordingly. For both the 2021/2022 and 2022/2023 seasons, the WHO recommended that quadrivalent egg‐based vaccines for use in the northern hemisphere influenza season contain the following strains: an A/Victoria/2570/2019 (H1N1)pdm09–like virus; an A/Cambodia/e0826360/2020 (H3N2)–like virus for the 2021/2022 season and an A/Darwin/9/2021 (H3N2)–like virus for the 2022/2023 season; a B/Washington/02/2019 (B/Victoria lineage)–like virus; a B/Phuket/3073/2013 (B/Yamagata lineage)–like virus for the 2021/2022 season and a B/Austria/1359417/2021 (B/Victoria lineage)–like virus for the 2022/2023 season; and a B/Phuket/3073/2013 (B/Yamagata lineage)–like virus. For both seasons, the circulating strains antigenically matched with the vaccine strains, although the matching was not perfect, especially for certain subclades of A(H3N2) [18]. Following the disappearance of the Yamagata lineage Type B virus since 2020 (and the COVID‐19 pandemic) and new expert recommendations [19], trivalent vaccines are recommended for the 2025/2026 influenza season [20]. Repeating the study across two seasons enables evaluation under varying vaccine formulations in this moving epidemiological context.

Our results also showed significantly fewer influenza‐related hospitalizations following HD than SD vaccination, with the rVE particularly high in 85+, and maintained across all age groups. These findings suggest that one‐third of this vulnerable population could have substantially benefited from HD vaccination and improved protection against influenza‐related hospitalizations.

To contextualize our results, in the present study, the IRR for influenza‐related hospitalizations among SD recipients was estimated at 173 for 100,000 person‐years. Assuming 8 million people aged 65+ received SDs during the 2022/2023 influenza vaccination campaign, this corresponds to approximately 12,000 influenza‐related hospitalizations. With an rVE of 27%, replacing SDs by HD in this population could have prevented about 3300 additional influenza‐related hospitalizations over the few weeks of the season. A French study covering eight epidemic seasons (11/2010 to 18/2017) [21] reported a median hospital length of 8 days for patients aged 65–84 years and 10 days for 85+, suggesting that these 3300 hospitalizations represent nearly 29,000 hospital days that could have been avoided using HD. This estimate excludes indirect costs and posthospitalization consequences in older people. For the 2022/2023 season, the vaccine coverage rate in 65+ was 54% [22]; a better vaccine coverage rate would have reduced hospitalizations further.

Our study had some limitations. Most of them were discussed in the article by Bricout et al. [10]. For example, we used the pharmacy dispensing date as a proxy for the vaccination date, whereas about 50% of patients were not vaccinated at the pharmacy (see Table S1). However, a sensitivity analysis (Supporting Information) using the first medical encounter within 2 weeks of pharmacy collection instead of pharmacy dispensing data did not alter the study findings (data not shown). More specifically, the rate of matching in DRIVEN Season 2 was lower than for Season 1 (70.1% vs. 99.2%), and 40% of 85+ HD recipients were not matched. Several reasons may explain these two observations, which are probably interrelated. Nearly twice as many HD doses were available on the French market in the 2022/2023 season compared with the 2021/2022 season. Therefore, as the matching ratio remained the same for both seasons, it was more difficult—and sometimes impossible—to find four controls per person within Season 2. If new studies were to be conducted for the 2023/2024 season or in countries where HD is more widely used, matching ratios should be reconsidered. Moreover, finding four controls fulfilling the matching criterion related to the index date (week of vaccination) was particularly difficult, as the HD was mainly dispensed at the beginning of the season, whereas the SD was consistently dispensed during the first weeks. Several hypotheses may explain the early dispensation of HD: Pharmacists knew that the vaccine was specifically designed for older adults and therefore prioritized it for the benefit of their older patients; people had heard about this vaccine and were requesting it from their pharmacists; in France, all influenza vaccines are fully reimbursed once they are recommended; pharmacists may have wanted to sell their stock of HD vaccines first since HD was more expensive for them to purchase; and the older the individuals or the more comorbidities they had, the earlier they tended to get vaccinated. This latest hypothesis was partially supported by vaccine distribution data, which showed 63.87% of the doses of influenza vaccine were dispensed within the first 4 weeks of the vaccination campaign in the 85+ compared with 58.15% and 54.30% in the 75–85 and 65–75 groups, respectively (Table S3). Unmatched HD recipients were older and frailer than matched HD recipients. This indication bias possibly affected the effectiveness of the HD and SDs, but DRIVEN was aimed at measuring the rVE and not the absolute effectiveness of each vaccine. Conversely, this bias and the fact that the rVE was higher in the 85+ than in the 65–75 or 75–85 tended to indicate that the difference in effectiveness reported between the HD and SDs was more perceptible in the oldest and most immunosenescent, and the indication bias possibly decreased the global rVE of the HD for the 2022/2023 season.

Our study had several strengths. DRIVEN Season 2 was conducted across a large sample (> 2 million people), capturing all HD provided free of charge in community pharmacies in France, giving a comprehensive overview of vaccine effectiveness. The large size of the study also allowed for subgroup analysis based on the age of recipients. It is to be noted that the maintenance of the usual 5% significance level for tests and the large sample size requires interpreting small statistically significant effects with caution. In addition, we conducted numerous sensitivity analyses and a falsification analysis to support the results of the main analysis. Finally, since the COVID‐19 pandemic, the number of PCR tests in cases of suspected respiratory tract infection has increased, thereby improving the specificity of influenza coding at hospital discharge, and consecutively allowing for more accurate identification of influenza cases for the influenza hospitalization outcomes.

## 5 Conclusions

This study provided the first published rVE data comparing HD vs. SDs for the season 2022–2023 in France. It found that HD provided better protection against influenza‐related hospitalizations compared with SDs in older people in a real‐world setting across all age groups (65–75, 75–85, and > 85 years). Its results were consistent with those reported for the 2021/2022 influenza season, but more robust given the larger number of HD recipients. These findings add to the six randomized controlled trials and 11 seasons of observational data showing the greater benefit of HD over SDs. During the 2022/2023 influenza season, in France, replacing SDs with HD would have prevented about 29,000 hospital days. Finally, these data support the decision of health authorities who recommend using HD in first intent or preferentially in older adults [23, 24, 25].

## Author Contributions

**Hélène Bricout:** conceptualization, methodology, formal analysis, writing – review and editing, writing – original draft. **Marie‐Cécile Levant:** conceptualization, methodology, formal analysis, writing – original draft, writing – review and editing. **Pascal Crépey:** conceptualization, methodology, formal analysis, writing – review and editing. **Gaëtan Gavazzi:** conceptualization, methodology, formal analysis, writing – review and editing. **Jacques Gaillat:** conceptualization, methodology, formal analysis, writing – review and editing. **Marine Dufournet:** conceptualization, methodology, formal analysis, writing – review and editing. **Nada Assi:** conceptualization, methodology, formal analysis, writing – review and editing. **Benjamin Grenier:** conceptualization, methodology, formal analysis, writing – review and editing. **Fanny Raguideau:** conceptualization, methodology, formal analysis, writing – review and editing. **Fabienne Péretz:** conceptualization, methodology, formal analysis, writing – review and editing. **Camille Salamand:** conceptualization, methodology, formal analysis, writing – review and editing. **Anne Mosnier:** conceptualization, methodology, formal analysis, writing – review and editing. **Laurence Watier:** conceptualization, methodology, formal analysis, writing – review and editing. **Odile Launay:** conceptualization, methodology, formal analysis, writing – review and editing. **Matthew M. Loiacono:** conceptualization, methodology, formal analysis, writing – original draft, writing – review and editing.

## Ethics Statement

The authors confirm that all relevant ethical guidelines have been followed, and any necessary IRB and/or ethics committee approvals have been obtained. In accordance with the regulations in force, the study protocol has been submitted to the Ethics and Scientific Committee for Research, Studies, and Evaluations in the field of health (CESREES, *Comité Ethique et Scientifique pour les Recherches, les Etudes et les Evaluations dans le domaine de la Santé*). The study protocol obtained three consecutive authorizations from the French data protection authority (CNIL, *Commission Nationale de l'Informatique et des Libertés*): Initial authorization, Decision No. DR‐2022‐049; Substantial modifications authorization, Decision DR‐2023‐013; Second substantial modifications authorization, Decision DR‐2023‐160.

## Conflicts of Interest

H.B., M.‐C.L., M.D., C.S., and M.M.L. are Sanofi employees and may hold shares in the company. N.A., B.G., and F.R. are HEVA employees, who received funding from Sanofi to run the study. P.C. reports to have participated in advisory committees organized by Sanofi and being a consultant for Sanofi. J.G. reports to have participated in advisory committees organized by GSK, MSD, Pfizer, and Sanofi. G.G. reports to have participated in advisory committees organized by Astellas, AstraZeneca, BioMerieux, MSD, Pfizer, Sanofi, Sanofi Pasteur, Sanofi Pasteur‐MSD, and Vifor; acted as a consultant and speaker for these companies; and participated in congresses on invitation by Eisai, MSD, Novartis, Pfizer, Sanofi, and Vifor. A.M. reports to have participated in advisory committees organized by Sanofi, Seqirus, and Viatris; to be a speaker for Sanofi, Seqirus, Viatris, and Novavax; and to be a member of the scientific board of the SFM (formerly known as GEIG). F.P. from Abelia Science provided medical writing support, which was funded by Sanofi. L.W. has received consulting fees from HEVA, Sanofi, and Pfizer for works outside the submitted work. O.L. reports to be a principal investigator in vaccine trials sponsored by Sanofi, MSD, Pfizer, GSK, and Moderna. She received financial support for travel to medical congress and personal fees for participation in advisory boards for Sanofi, MSD, Pfizer, and GSK.

## Supporting information


**Table S1:** Medication codes, ICD‐10 codes, and references used during the study.

**Table S2:** Characteristics of community‐dwelling 65+ HD or SD recipients during the 2022/2023 season (*N* = 7,914,298). HD: high‐dose influenza vaccine; SD: standard‐dose influenza vaccine.

**Table S3:** Characteristics of community‐dwelling 65+ influenza‐vaccine recipients during the 2022/2023 season, by age group (*N* = 7,914,298).

**Table S4:** Characteristics of matched and unmatched 65+ HD or SD recipients during the 2022/2023 season (*N* = 7,914,298). HD: high‐dose influenza vaccine; SD: standard‐dose influenza vaccine.

**Data S1:** Description of stability analysis.

**Figure S1:** HD and SD distribution over the influenza vaccine campaign (2022/2023). HD: high‐dose influenza vaccine; SD: standard‐dose influenza vaccine.

## Data Availability

Restrictions apply to the availability of the data supporting the study findings, as they contain potentially identifying and sensitive patient information. These data cannot be shared publicly as they are part of the National Health Data System (SNDS, *Système national des Données de Santé*). They are available from the HDH (Health Data Hub; https://www.health‐data‐hub.fr/). Special permission to access the data for this study was granted by the Ethics and Scientific Committee for Research, Studies, and Evaluations in the Field of Health (CESREES, *Comité Ethique et Scientifique pour les Recherches, les Etudes et les Evaluations dans le domaine de la Santé*).
