# Supplementary material for: Relative Effectiveness of High‐Dose vs. Standard‐Dose Influenza Vaccines in Preventing Hospitalizations: A National Retrospective Cohort Study in France, 2022/2023 Season
Source: Influenza Other Respir Viruses. 2025 Nov 16;19(11):e70193. doi: 10.1111/irv.70193 (PMC12620122; doi:10.1111/irv.70193)
Supplement: Supplementary file 1 — Table S1: Medication codes, ICD‐10 codes, and references used during the study. [file IRV-19-e70193-s001.docx]

**Medication codes, ICD-10 codes, and references used during the study**

| Medication codes (UCD13 and CIP13) for SD and HD | | | | | |
| --- | --- | --- | --- | --- | --- |
| Vaccine | | **UCD13 code** | **CIP13 code** | | |
| Standard-dose | |  |  | | |
| - INFLUVAC TETRA SUSP INJ SER 0,5ML | | 3400894338703 | 3400930117712 | | |
| - VAXIGRIPTETRA SUSP INJ SER 0,5ML | | 3400894329657 | 3400928099877 | | |
|  | | 3400894329657 | 3400930067727 | | |
| High-Dose | |  |  | | |
| - EFLUELDA SUSP INJ SER VACCIN | | 3400890004602 | 3400930205372 | | |
|  |  |  | 3400930205389 | | |
|  |  |  | 3400930205426 | | |
|  |  |  | 3400930205402 | | |
|  |  |  | 3400930205396 | | |
|  |  |  | 3400930205419 | | |
| ICD-10 discharge codes for hospitalization | | | |  |  |
| Definition | **ICD-10 codes** | | |  |  |
| Influenza hospitalization | J09 to J11 | | |  |  |
| Pneumonia hospitalization | J12 to J18 | | |  |  |
| Respiratory hospitalization | J00 to J06, J09 to J18, J40-J41, J96 | | |  |  |
| Cardiovascular hospitalization | I16, I20 to I22, I24, I24, I26, I30, I40, I46 to I50, I63, I65-I66, G45-G46, J96 | | |  |  |
| Cardiorespiratory hospitalization | J00 to J06, J09 to J18, J40-J41, J96  I20 to I22, I24, I24, I26, I30, I40, I46 to I50, I63, I65-I66, G45-G46 | | |  |  |
| COVID-19 hospitalization | U071, U0710, U0711, U0714, U0715 | | |  |  |
| Urinary tract infection hospitalization | N410, N412-N413, N418, N419, N10, N110, N12, N136, N300, N309, T835 | | |  |  |
| Erysipelas hospitalization | A46 | | |  |  |
| Cataract hospitalization | BFGA427, BFGA004, BFPA002, BFGA368, BFGA008, BFGA002, BFGA003, BFGA010, BFGA006, GFGA009 | | |  |  |
| ICD-10 codes or references used to identify comorbidities | | | | |  |
| Comorbidity | **Codes or references** | | | |  |
| Diabetes | Rachas A, Gastaldi-Menager C, Denis P, Barthelemy P, Constantinou P, Drouin J, et al. The Economic Burden of Disease in France From the National Health Insurance Perspective: The Healthcare Expenditures and Conditions Mapping Used to Prepare the French Social Security Funding Act and the Public Health Act. Med Care. 2022;60(9):655-64. | | | |  |
| Obesity and/or history of obesity surgery | HFCA001, HFCC003, HFFA001, HFFA011, HFFC004, HFFC018, HFGC900, HFKA001, HFKA002, HFKC001, HFLC900, HFLE002, HFMA009, HFMA010, HFMA011, HFMC006, HFMC007, HFMC008, HGCA009, HGCC027, E66 | | | |  |
| Undernourishment/or history of undernourishment | E43, E44, E46 | | | |  |
| COPD/Asthma | Rachas A, Gastaldi-Menager C, Denis P, Barthelemy P, Constantinou P, Drouin J, et al. The Economic Burden of Disease in France From the National Health Insurance Perspective: The Healthcare Expenditures and Conditions Mapping Used to Prepare the French Social Security Funding Act and the Public Health Act. Med Care. 2022;60(9):655-64. | | | |  |
| Dementia | Rachas A, Gastaldi-Menager C, Denis P, Barthelemy P, Constantinou P, Drouin J, et al. The Economic Burden of Disease in France From the National Health Insurance Perspective: The Healthcare Expenditures and Conditions Mapping Used to Prepare the French Social Security Funding Act and the Public Health Act. Med Care. 2022;60(9):655-64. | | | |  |
| Cardiovascular diseases | Rachas A, Gastaldi-Menager C, Denis P, Barthelemy P, Constantinou P, Drouin J, et al. The Economic Burden of Disease in France From the National Health Insurance Perspective: The Healthcare Expenditures and Conditions Mapping Used to Prepare the French Social Security Funding Act and the Public Health Act. Med Care. 2022;60(9):655-64. | | | |  |
| Immunocompromised individuals | Rachas A, Gastaldi-Menager C, Denis P, Barthelemy P, Constantinou P, Drouin J, et al. The Economic Burden of Disease in France From the National Health Insurance Perspective: The Healthcare Expenditures and Conditions Mapping Used to Prepare the French Social Security Funding Act and the Public Health Act. Med Care. 2022;60(9):655-64.  And  Wyplosz B, Fernandes J, Goussiaume G, Moïsi J, Lortet-Tieulent J, Vainchtock A, et al. Adults at risk of pneumococcal disease in France. Infect Dis Now. 2021 Nov;51(8):661–6. | | | |  |
| Chronic liver disease | Rachas A, Gastaldi-Menager C, Denis P, Barthelemy P, Constantinou P, Drouin J, et al. The Economic Burden of Disease in France From the National Health Insurance Perspective: The Healthcare Expenditures and Conditions Mapping Used to Prepare the French Social Security Funding Act and the Public Health Act. Med Care. 2022;60(9):655-64. | | | |  |
| Terminal chronic kidney failure | Rachas A, Gastaldi-Menager C, Denis P, Barthelemy P, Constantinou P, Drouin J, et al. The Economic Burden of Disease in France From the National Health Insurance Perspective: The Healthcare Expenditures and Conditions Mapping Used to Prepare the French Social Security Funding Act and the Public Health Act. Med Care. 2022;60(9):655-64. | | | |  |

HD: high-dose influenza vaccine; SD: standard-dose influenza vaccine
