# Supplementary material for: Relative Effectiveness of High‐Dose vs. Standard‐Dose Influenza Vaccines in Preventing Hospitalizations: A National Retrospective Cohort Study in France, 2022/2023 Season
Source: Influenza Other Respir Viruses. 2025 Nov 16;19(11):e70193. doi: 10.1111/irv.70193 (PMC12620122; doi:10.1111/irv.70193)
Supplement: Supplementary file 5 — Data S1: Description of stability analysis. [file IRV-19-e70193-s006.docx]

**Supplementary Materials for:**

**Relative effectiveness of high-dose vs standard-dose influenza vaccines in preventing hospitalizations: a national retrospective cohort study in France, 2022/23 season**

1. **Data Sources**

The SNDS collates health data collected by NHI agencies. We used data from two pre-existing databases: the NHI Système National d'Information Inter-Régimes de l'Assurance Maladie (SNIIRAM) and the hospital discharge database (Programme de Médicalisation des Systèmes d'Information; PMSI). The SNDS contains individual-level data for outpatient and private healthcare facilities health expenditure billing for reimbursement purposes from the SNIIRAM that are linked to the hospitalisation database PMSI using a unique, anonymous identifier, the social security number (Numéro d'inscription au répertoire de l'INSEE; NIR). All subjects have years of history of healthcare consumption within the database as the healthcare coverage is mandatory and free in France.

In the French health care system, individuals who are eligible for influenza vaccination receive a voucher for their influenza vaccine to be dispensed at a pharmacy and then administered by a pharmacist, general practitioner (GP) or nurse (as GP surgeries in France do not store vaccines themselves). We assumed the date of vaccination (“index date”) was approximated by the date of influenza vaccine pharmacy dispensation.

1. **Propensity score matching**

Propensity score (PS) matching was performed using a greedy matching algorithm without replacement with a caliper value equal to 20% of the variability (standard deviation [STD]) of the logarithm of the propensity score values (0.2*STD(log(PS)), with an exact matching constraint on sex, age group (65 to 75 years of age, 75 to 85 years of age, and over 85 years of age), geographical region and week of vaccine dispensing. Geographical region and dispensing week were included to address temporal and geographical gradients likely associated with access to HD-QIV (e.g., supply) and influenza virus exposure (i.e., viral activity and circulation). The propensity score was computed using a logistic regression model with vaccine group as the dependent variable and all relevant covariates as independent variables. These independent variables included: number of GP consultations in the past 12 months, number of all-cause hospitalizations in the past 12 months, number of influenza vaccinations (in the five previous seasons), age (continuous, and in categories [used for the exact constraint]: 65-75 years, 75-85 years, above 85 years), sex, vaccine administration setting (either at pharmacy or not), quintiles of the French social deprivation index (FDep), vaccination status for influenza in the preceding season (HD-QIV, SD-QIV, not vaccinated), COVID-19 vaccination status (completed, on-going [i.e., partial], unvaccinated), pneumonia vaccination status in the past five years, geographical region, and a diagnosis in the previous five years for diabetes, obesity and/or history of obesity surgery (referred to herein as “obesity”), undernourishment or history of undernourishment (referred to herein as “malnutrition”), chronic respiratory diseases, dementia, myocardial infarction, chronic coronary disease, chronic heart failure, peripheral artery disease (PAD), haematological tumours, solid tumours, solid organ transplant, stem cells transplant, HIV (human immunodeficiency virus), drug-induced immunosuppression, chronic liver diseases and severe renal disease, and number of comorbidities of interest.

The quality of the matching was assessed by computing standardized mean differences (SMD) before and after matching. An absolute value of SMD below the cutoff of 0.1 is indicative of a good balance achieved for the covariates [Ref.1, Ref.2]. In our analysis, SMD absolute values were all below the 0.1threshold, the estimation models for IRR and rVE were thus unadjusted and required no further double robust methods to be implemented.

Ref.1- Stuart EA, Lee BK, Leacy FP. Prognostic score-based balance measures can be a useful diagnostic for propensity score methods in comparative effectiveness research. J Clin Epidemiol. 2013 Aug;66(8 Suppl):S84-S90.e1.

Ref.2- Zhang Z, Kim HJ, Lonjon G, Zhu Y. Balance diagnostics after propensity score matching. Ann Transl Med. 2019 Jan;7(1):16.

1. **Inverse probability of treatment weighting (IPTW) and multivariate regression**

To assess consistency and robustness of the results, two different stability analyses were performed:

- One using Inverse Probability of Treatment Weighting (IPTW).
- An additional using classical multivariable regression.

**The results from the IPTW analysis** were clinically implausible and a falsification analysis using IPTW indicated large residual confounding.

This likely reflects issues with the positivity assumption, which requires both exposed and unexposed individuals at each level of every confounder. Violating this assumption can disrupt the IPTW methodology and hinder causal interpretation of the treatment effect [Ref.1].

Unlike the propensity score matching method, which excludes unmatched HD recipients from the analysis, IPTW retains them in the analysis. However, a significant proportion of these individuals are assigned extreme weights, when they exhibit substantial differences in confounder distribution (i.e., when their propensity score is close to 0 or 1), making the method highly sensitive to violations of the positivity assumption. Therefore, trimming observations with extreme weights is often recommended when using IPTW [Ref.2].

Since only 71.7% of HD recipients could be matched to SD recipients, the positivity assumption appears to be violated. Even subsequent to the computation of stabilized weights, rVE results remained significantly influenced by extreme weights. Following the trimming to the 5th and 95th percentiles, the IPTW results became plausible and aligned with the main analysis results.

**The results from the multivariate regression analysis** (all, matched and unmatched recipients; for all results) were consistent with the main analysis findings.

The following tables present results obtained using the PD code to ascertain hospitalization diagnosis (PD only) and after exclusion of hospitalizations with a discharge diagnosis code associated with COVID-19 (COVID-19 excluded).

Ref.1- Chesnaye NC, Stel VS, Tripepi G, Dekker FW, Fu EL, Zoccali C, Jager KJ. An introduction to inverse probability of treatment weighting in observational research. Clin Kidney J. 2021 Aug 26;15(1):14-20. doi: 10.1093/ckj/sfab158. PMID: 35035932; PMCID: PMC8757413.

Ref.2- Koichiro Shiba, Takuya Kawahara, Using Propensity Scores for Causal Inference: Pitfalls and Tips, Journal of Epidemiology, 2021, Volume 31, Issue 8, Pages 457-463, Released on J-STAGE August 05, 2021, Advance online publication June 12, 2021, Online ISSN 1349-9092, Print ISSN 0917-5040, <https://doi.org/10.2188/jea.JE20210145>.

Hospitalizations were ascertained by the International Classification of Diseases 10th Revision (ICD-10) discharge diagnosis code. The ICD-10 discharge diagnosis code in the database could be noted as primary (PD), related (RD), or associated (AD).

**Full cohort (PD only, covid excluded)**

| Examined outcome | QIV vaccine group | Number of events | Person-years | Crude incidence rate for 100,000 person-years (95% CI) | aRR (95%CI) | arVE (95%CI) | P-value | Bonferonni-Holm P-value |
| --- | --- | --- | --- | --- | --- | --- | --- | --- |
| Nb of P/I hospitalizations | HD QIV | 7,184 | 577,125.59 | 1,244.79 (1,216.34-1,273.91) | 0.97 [0.94;1.00] | 2.74 [-0.26;5.64] | 0.0727 | 0.3637 |
|  | SD QIV | 39,745 | 3,945,925.30 | 1,007.24 (997.39-1,017.19) | 1.00 | . | 0.0727 | 0.3637 |
| Nb of P/I hospitalizations (<75 years) | HD QIV | 1,310 | 230,605.29 | 568.07 (538.13-599.68) | 0.99 [0.92;1.06] | 1.26 [-5.65;7.72] | 0.7129 | . |
|  | SD QIV | 9,720 | 1,962,013 | 495.41 (485.66-505.36) | 1.00 | . | 0.7129 | . |
| Nb of P/I hospitalizations (75-85 years) | HD QIV | 2,823 | 240,613.82 | 1,173.25 (1,130.76-1,217.34) | 1.00 [0.95;1.04] | 0.48 [-4.39;5.12] | 0.8434 | . |
|  | SD QIV | 15,451 | 1,469,299.10 | 1,051.60 (1,035.15-1,068.31) | 1.00 | . | 0.8434 | . |
| Nb of P/I hospitalizations (>85 years) | HD QIV | 3,051 | 105,906.48 | 2,880.84 (2,780.42-2,984.90) | 0.95 [0.90;0.99] | 5.25 [0.72;9.58] | 0.0237 | . |
|  | SD QIV | 14,574 | 514,613.19 | 2,832.03 (2,786.42-2,878.38) | 1.00 | . | 0.0237 | . |
| Nb of influenza hospitalizations | HD QIV | 874 | 577,125.59 | 151.44 (141.73-161.82) | 0.73 [0.67;0.79] | 27.42 [21.23;33.13] | <.0001 | <.0001 |
|  | SD QIV | 6,103 | 3,945,925.30 | 154.67 (150.83-158.60) | 1.00 | . | <.0001 | <.0001 |
| Nb of pneumonia hospitalizations | HD QIV | 6,310 | 577,125.59 | 1,093.35 (1,066.71-1,120.67) | 1.02 [0.99;1.05] | -2.05 [-5.41;1.20] | 0.2187 | 0.6562 |
|  | SD QIV | 33,642 | 3,945,925.30 | 852.58 (843.51-861.73) | 1.00 | . | 0.2187 | 0.6562 |
| Nb of respiratory disease hospitalizations | HD QIV | 8,523 | 577,125.59 | 1,476.80 (1,445.78-1,508.49) | 0.99 [0.96;1.02] | 1.02 [-1.81;3.77] | 0.4747 | 0.7783 |
|  | SD QIV | 46,643 | 3,945,925.30 | 1,182.06 (1,171.38-1,192.83) | 1.00 | . | 0.4747 | 0.7783 |
| Nb of cardiovascular hospitalizations | HD QIV | 24,971 | 577,125.59 | 4,326.80 (4,273.47-4,380.80) | 1.02 [1.00;1.03] | -1.51 [-3.26;0.21] | 0.0858 | 0.3637 |
|  | SD QIV | 143,378 | 3,945,925.30 | 3,633.57 (3,614.81-3,652.43) | 1.00 | . | 0.0858 | 0.3637 |
| Nb of cardiorespiratory hospitalizations | HD QIV | 32,230 | 577,125.59 | 5,584.58 (5,523.94-5,645.88) | 1.01 [0.99;1.02] | -0.67 [-2.20;0.84] | 0.3892 | 0.7783 |
|  | SD QIV | 183,638 | 3,945,925.30 | 4,653.87 (4,632.64-4,675.21) | 1.00 | . | 0.3892 | 0.7783 |

HD: high dose; SD: standard dose

**Matched cohorts 1:4 (PD only, covid excluded)**

| Examined outcome | QIV vaccine group | Number of events | Person-years | Crude incidence rate for 100,000 person-years (95% CI) | aRR (95%CI) | arVE (95%CI) | P-value | Bonferonni-Holm P-value |
| --- | --- | --- | --- | --- | --- | --- | --- | --- |
| Nb of P/I hospitalizations | HD QIV | 4,420 | 410,295.93 | 1,077.28 (1,045.98-1,109.51) | 0.96 [0.93;1.00] | 3.85 [0.22;7.33] | 0.0376 | 0.1504 |
|  | SD QIV | 18,236 | 1,642,211 | 1,110.46 (1,094.46-1,126.69) | 1.00 | . | 0.0376 | 0.1504 |
| Nb of P/I hospitalizations (<75 years) | HD QIV | 996 | 185,975.43 | 535.55 (503.31-569.87) | 0.97 [0.89;1.04] | 3.47 [-4.25;10.62] | 0.3679 | . |
|  | SD QIV | 4,037 | 744,039.99 | 542.58 (526.10-559.58) | 1.00 | . | 0.3679 | . |
| Nb of P/I hospitalizations (75-85 years) | HD QIV | 1,730 | 161,084.30 | 1,073.98 (1,024.54-1,125.80) | 0.98 [0.92;1.04] | 2.09 [-3.83;7.68] | 0.4805 | . |
|  | SD QIV | 7,031 | 644,689.56 | 1,090.61 (1,065.41-1,116.40) | 1.00 | . | 0.4805 | . |
| Nb of P/I hospitalizations (>85 years) | HD QIV | 1,694 | 63,236.20 | 2,678.85 (2,554.27-2,809.49) | 0.94 [0.89;1.00] | 5.73 [-0.05;11.17] | 0.0518 | . |
|  | SD QIV | 7,168 | 253,481.50 | 2,827.82 (2,763.11-2,894.04) | 1.00 | . | 0.0518 | . |
| Nb of influenza hospitalizations | HD QIV | 516 | 410,295.93 | 125.76 (115.37-137.10) | 0.71 [0.65;0.79] | 28.62 [21.00;35.50] | <.0001 | <.0001 |
|  | SD QIV | 2,847 | 1,642,211 | 173.36 (167.11-179.85) | 1.00 | . | <.0001 | <.0001 |
| Nb of pneumonia hospitalizations | HD QIV | 3,904 | 410,295.93 | 951.51 (922.12-981.83) | 1.01 [0.97;1.05] | -0.86 [-4.92;3.05] | 0.6713 | 0.6713 |
|  | SD QIV | 15,389 | 1,642,211 | 937.09 (922.40-952.01) | 1.00 | . | 0.6713 | 0.6713 |
| Nb of respiratory disease hospitalizations | HD QIV | 5,287 | 410,295.93 | 1,288.58 (1,254.31-1,323.79) | 0.98 [0.95;1.02] | 1.80 [-1.63;5.10] | 0.3000 | 0.6512 |
|  | SD QIV | 21,381 | 1,642,211 | 1,301.97 (1,284.63-1,319.53) | 1.00 | . | 0.3000 | 0.6512 |
| Nb of cardiovascular hospitalizations | HD QIV | 16,424 | 410,295.93 | 4,002.98 (3,942.23-4,064.67) | 1.02 [1.00;1.04] | -2.38 [-4.49;-0.30] | 0.0245 | 0.1223 |
|  | SD QIV | 63,951 | 1,642,211 | 3,894.22 (3,864.16-3,924.52) | 1.00 | . | 0.0245 | 0.1223 |
| Nb of cardiorespiratory hospitalizations | HD QIV | 20,895 | 410,295.93 | 5,092.67 (5,024.09-5,162.19) | 1.01 [0.99;1.03] | -1.15 [-3.00;0.67] | 0.2171 | 0.6512 |
|  | SD QIV | 82,393 | 1,642,211 | 5,017.21 (4,983.07-5,051.58) | 1.00 | . | 0.2171 | 0.6512 |

**IPTW individuals that are not included in the 1:4 matching ratio (PD only, covid excluded)**

| **Examined outcome** | **QIV vaccine group** | **Number of events** | **Person-years** | **Crude incidence rate for 100,000 person-years (95% CI)** | **aRR (95%CI)** | **arVE (95%CI)** | **P-value** | **Bonferonni-Holm P-value** |
| --- | --- | --- | --- | --- | --- | --- | --- | --- |
| Nb of P/I hospitalizations | HD QIV | 2,764.00 | 166,829.65 | 1,656.78 (1,596.15-1,719.71) | 0.99 [0.94;1.05] | 0.70 [-5.04;6.14] | 0.8058 | 1.0000 |
|  | SD QIV | 21,509.00 | 2,303,714.30 | 933.67 (921.27-946.23) | 1.00 | . | 0.8058 | 1.0000 |
| Nb of P/I hospitalizations  (<75 years) | HD QIV | 314.00 | 44,629.86 | 703.56 (629.90-785.85) | 1.07 [0.91;1.26] | -6.70 [-25.61;9.36] | 0.4356 | . |
|  | SD QIV | 5,683 | 1,217,973 | 466.60 (454.62-478.89) | 1.00 | . | 0.4356 | . |
| Nb of P/I hospitalizations  (75-85 years) | HD QIV | 1,093.00 | 79,529.52 | 1,374.33 (1,295.23-1,458.27) | 1.03 [0.94;1.12] | -2.94 [-12.36;5.69] | 0.5166 | . |
|  | SD QIV | 8,420.00 | 824,609.52 | 1,021.09 (999.51-1,043.13) | 1.00 | . | 0.5166 | . |
| Nb of P/I hospitalizations  (>85 years) | HD QIV | 1,357.00 | 42,670.27 | 3,180.20 (3,015.42-3,353.98) | 0.96 [0.88;1.03] | 4.37 [-3.49;11.64] | 0.2675 | . |
|  | SD QIV | 7,406.00 | 261,131.69 | 2,836.12 (2,772.26-2,901.45) | 1.00 | . | 0.2675 | . |
| Nb of influenza  hospitalizations | HD QIV | 358.00 | 166,829.65 | 214.59 (193.47-238.01) | 0.74 [0.64;0.86] | 25.96 [14.38;35.98] | <.0001 | 0.0003 |
|  | SD QIV | 3,256.00 | 2,303,714.30 | 141.34 (136.57-146.28) | 1.00 | . | <.0001 | 0.0003 |
| Nb of pneumonia  hospitalizations | HD QIV | 2,406.00 | 166,829.65 | 1,442.19 (1,385.70-1,500.98) | 1.04 [0.98;1.11] | -4.49 [-11.00;1.64] | 0.1543 | 0.7717 |
|  | SD QIV | 18,253.00 | 2,303,714.30 | 792.33 (780.92-803.91) | 1.00 | . | 0.1543 | 0.7717 |
| Nb of respiratory disease  hospitalizations | HD QIV | 3,236.00 | 166,829.65 | 1,939.70 (1,874.01-2,007.70) | 1.01 [0.96;1.06] | -0.70 [-6.16;4.47] | 0.7942 | 1.0000 |
|  | SD QIV | 25,262.00 | 2,303,714.30 | 1,096.58 (1,083.14-1,110.18) | 1.00 | . | 0.7942 | 1.0000 |
| Nb of cardiovascular  hospitalizations | HD QIV | 8,547.00 | 166,829.65 | 5,123.20 (5,015.73-5,232.96) | 0.99 [0.96;1.02] | 0.94 [-2.43;4.19] | 0.5810 | 1.0000 |
|  | SD QIV | 79,427.00 | 2,303,714.30 | 3,447.78 (3,423.89-3,471.84) | 1.00 | . | 0.5810 | 1.0000 |
| Nb of cardiorespiratory  hospitalizations | HD QIV | 11,335.00 | 166,829.65 | 6,794.36 (6,670.43-6,920.58) | 0.99 [0.96;1.02] | 0.75 [-2.21;3.62] | 0.6167 | 1.0000 |
|  | SD QIV | 101,245.00 | 2,303,714.30 | 4,394.88 (4,367.89-4,422.03) | 1.00 | . | 0.6167 | 1.0000 |

1. **ICD-10 codes or references to the codes used to identify comorbidities**

| Comorbity | Codes or references |
| --- | --- |
| Diabetes | Rachas A, Gastaldi-Menager C, Denis P, Barthelemy P, Constantinou P, Drouin J, et al. The Economic Burden of Disease in France From the National Health Insurance Perspective: The Healthcare Expenditures and Conditions Mapping Used to Prepare the French Social Security Funding Act and the Public Health Act. Med Care. 2022;60(9):655-64. |
| Obesity and/or history of obesity surgery | HFCA001, HFCC003, HFFA001, HFFA011, HFFC004, HFFC018, HFGC900, HFKA001, HFKA002, HFKC001, HFLC900, HFLE002, HFMA009, HFMA010, HFMA011, HFMC006, HFMC007, HFMC008, HGCA009, HGCC027, E66 |
| Undernourishment/or history of undernourishment | E43, E44, E46 |
| Chronic respiratory diseases | Rachas A, Gastaldi-Menager C, Denis P, Barthelemy P, Constantinou P, Drouin J, et al. The Economic Burden of Disease in France From the National Health Insurance Perspective: The Healthcare Expenditures and Conditions Mapping Used to Prepare the French Social Security Funding Act and the Public Health Act. Med Care. 2022;60(9):655-64. |
| Dementia | Rachas A, Gastaldi-Menager C, Denis P, Barthelemy P, Constantinou P, Drouin J, et al. The Economic Burden of Disease in France From the National Health Insurance Perspective: The Healthcare Expenditures and Conditions Mapping Used to Prepare the French Social Security Funding Act and the Public Health Act. Med Care. 2022;60(9):655-64. |
| Cardiovascular diseases (including myocardial infarction (MI), chronic coronary disease, chronic heart failure, Peripheral Artery disease (PAD)) | Rachas A, Gastaldi-Menager C, Denis P, Barthelemy P, Constantinou P, Drouin J, et al. The Economic Burden of Disease in France From the National Health Insurance Perspective: The Healthcare Expenditures and Conditions Mapping Used to Prepare the French Social Security Funding Act and the Public Health Act. Med Care. 2022;60(9):655-64. |
| Immunocompromised individuals (including cancer patients (solid and hematological tumours), organ/stem cells transplanted patients, HIV patients, and chronic autoimmune or inflammatory diseases treated with immunosuppressive or biologic drugs) | Rachas A, Gastaldi-Menager C, Denis P, Barthelemy P, Constantinou P, Drouin J, et al. The Economic Burden of Disease in France From the National Health Insurance Perspective: The Healthcare Expenditures and Conditions Mapping Used to Prepare the French Social Security Funding Act and the Public Health Act. Med Care. 2022;60(9):655-64.  And  Wyplosz B, Fernandes J, Goussiaume G, Moïsi J, Lortet-Tieulent J, Vainchtock A, et al. Adults at risk of pneumococcal disease in France. Infect Dis Now. 2021 Nov;51(8):661–6. |
| Chronic liver disease | Rachas A, Gastaldi-Menager C, Denis P, Barthelemy P, Constantinou P, Drouin J, et al. The Economic Burden of Disease in France From the National Health Insurance Perspective: The Healthcare Expenditures and Conditions Mapping Used to Prepare the French Social Security Funding Act and the Public Health Act. Med Care. 2022;60(9):655-64. |
| Terminal chronic kidney failure | Rachas A, Gastaldi-Menager C, Denis P, Barthelemy P, Constantinou P, Drouin J, et al. The Economic Burden of Disease in France From the National Health Insurance Perspective: The Healthcare Expenditures and Conditions Mapping Used to Prepare the French Social Security Funding Act and the Public Health Act. Med Care. 2022;60(9):655-64. |

1. **Sensitivity analysis**

A sensitivity analysis was conducted to consider the administration date of the vaccine instead of the dispensing date, for individuals that did not get administered in a pharmacy (1). In this analysis, the QIV administration date was defined using a proxy: the date of the first consultation with a general practitioner, midwife, or nurse within the two weeks following the dispensing of the vaccine.

For individuals vaccinated in a pharmacy, the starting point (time zero) for the analysis was 14 days after the administration codes registration. For other individuals, the starting point (time zero) for the analysis was 14 days after this proxy administration date, to account for the peak immune response to the vaccine.

It is to note that the median time between the date of dispensing and of the following medical visit (GP, midwife, nurse) was 2 days.

(1) Grave C, Boucheron P, Rudant J, Mikaeloff Y, Tubert-Bitter P, Escolano S, et al. Seasonal influenza vaccine and Guillain-Barré syndrome: A self-controlled case series study. Neurology. 2020 May 19;94(20):e2168–79.
