# Supplementary figures and images for: Relative Effectiveness of High‐Dose vs. Standard‐Dose Influenza Vaccines in Preventing Hospitalizations: A National Retrospective Cohort Study in France, 2022/2023 Season
Source: Influenza Other Respir Viruses. 2025 Nov 16;19(11):e70193. doi: 10.1111/irv.70193 (PMC12620122; doi:10.1111/irv.70193)

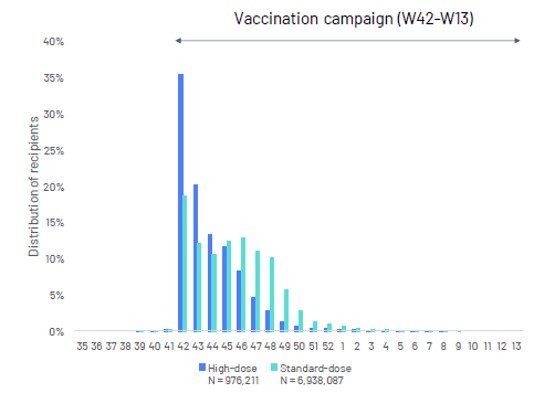

Supplement: Supplementary file 6 — Figure S1: HD and SD distribution over the influenza vaccine campaign (2022/2023). HD: high‐dose influenza vaccine; SD: standard‐dose influenza vaccine. [file IRV-19-e70193-s004.jpg]
